# Effects on intrusive memories of playing Tetris with or without simultaneous and deliberate memory recall

**DOI:** 10.1002/jts.70104

**Published:** 2026-07-01

**Authors:** Kevin van Schie, Henrik Kessler, Iris M. Engelhard

**Affiliations:** ^1^ Department of Medical and Clinical Psychology Tilburg University Tilburg The Netherlands; ^2^ Department of Psychosomatic Medicine and Psychotherapy Campus Fulda Philipps‐University of Marburg Marburg Germany; ^3^ Department of Clinical Psychology Faculty of Social and Behavioural Sciences Utrecht University Utrecht the Netherlands; ^4^ Department of Clinical Psychological Science Maastricht University Maastricht the Netherlands

## Abstract

Two interventions that tax visuospatial working memory (WM) may reduce intrusive memories despite differing procedurally: playing Tetris with or without memory recall. Accordingly, this study examined how engagement with negative memories influences intrusion frequency (Experiment 1) and WM load (Experiment 2). In Experiment 1 (*N* = 90), participants viewed a trauma film and were assigned to Tetris without memory recall, Tetris with memory recall, or a no‐task control condition. Participants rated memory vividness and unpleasantness before and after the intervention and recorded film‐related intrusions in a diary for 1 week. In Experiment 2 (*N* = 45), a reaction time task assessed whether brief memory reactivation (as used in the Tetris without memory recall condition) or concurrent, deliberate recall (as used in the Tetris with memory recall condition) differentially taxed WM. Experiment 1 showed that the Tetris with memory recall task led to the largest reductions in memory vividness, η_p_^2^ = .09, *p* = .017, and unpleasantness, η_p_^2^ = .09, *p* = .015, relative to the other conditions, but, unexpectedly, intrusion frequency did not differ between interventions, η_p_^2^ = .02, BF_01_ = 5.57, *p* = .448. Experiment 2 indicated that only simultaneous, deliberate recall imposed a WM load, η_p_^2^ = .11, *p* = .032. The lack of effects on intrusive memories warrants consideration of methodological differences between this and prior Tetris research and highlights the need to clarify which elements and mechanisms of the intervention—whether implemented with or without deliberate recall—modulate intrusions before translating these findings into clinical practice.

## EXPERIMENT 1

Most people experience a potentially traumatic event at least once in their life, and a minority of individuals develop posttraumatic stress disorder (PTSD; Santiago et al., 2013). PTSD is marked by persistent intrusions related to a traumatic event, avoidance, negative alterations in cognition and mood, and increased arousal or reactivity (American Psychiatric Association [APA], 2013). Intrusions typically take the form of distressing visual images (Ehlers et al., 2002). To mitigate the occurrence and impact of intrusive memories, several behavioral interventions have been tested (e.g., Holmes et al., 2009, 2010; Iyadurai et al., 2018).

One strategy for targeting intrusive memories involves selectively taxing visuospatial working memory (WM) shortly *after* exposure to analogue trauma (i.e., a standardized trauma film containing aversive content used in laboratory settings to model real‐life traumatic experiences). This is typically done using the computer game Tetris, which engages visuospatial WM resources through mental rotation (e.g., Hagenaars et al., 2017; Holmes et al., 2009, 2010). Holmes et al. (2010) hypothesized that this may block excessive perceptual processing of the traumatic event, thereby selectively disrupting the memory's sensory aspects that underlie intrusive images. Accordingly, playing Tetris after a trauma film has been shown to reduce intrusions compared to a no‐task control condition (e.g., Asselbergs et al., 2023; Holmes et al., 2009). In these studies that use trauma films, participants are typically first presented with a brief standardized visual cue to refocus on the analogue traumatic memory before playing Tetris consecutively.

An alternative dual‐task approach to target aversive or intrusive memories involves two tasks to be performed concurrently (Engelhard et al., 2010, 2019; Gunter & Bodner, 2008; van den Hout & Engelhard, 2012): deliberately recalling the memory “hotspot” while simultaneously engaging in a cognitively demanding secondary task, such as playing Tetris, making eye movements, or drawing a complex figure. Andrade et al. (1997) hypothesized that simultaneous memory recall and secondary task performance compete for limited WM resources, thereby disrupting memory retrieval and reducing memory vividness and/or unpleasantness. The resulting degraded memory may then be reconsolidated (van den Hout & Engelhard, 2012) or reappraised in a less distressing way (e.g., Engelhard et al., 2019). Indeed, vividness and unpleasantness have been shown to decrease when a task (e.g., making eye movements) is performed simultaneously with memory recall (Mertens et al., 2021) but not when it is performed after memory recall (Gunter & Bodner, 2008 [Experiment 1], but see van Schie & van Veen, 2023). These findings help explain the effects of making eye movements in eye movement desensitization and reprocessing (EMDR; Lee & Cuijpers, 2013; Shapiro, 2018). Furthermore, because PTSD intrusions often involve the hotspot (e.g., Grey & Holmes, 2008), reducing vividness and unpleasantness may also reduce intrusions.

Consecutive‐task interventions are often applied within 6 hr of experiencing (analogue) trauma exposure (Holmes et al., 2009, 2010), whereas dual‐task interventions are typically applied after the retrieval of autobiographical memories (Mertens et al., 2021). However, there are exceptions. Consecutive‐task interventions also reduce intrusions of existing memories in the days (Hagenaars et al., 2017; James et al., 2015; Kessler et al., 2020) or even weeks (Kessler et al., 2018) following trauma exposure. Similarly, dual‐task interventions can also reduce memory vividness and unpleasantness shortly after exposure to new materials, such as pictures or film clips (e.g., van den Hout et al., 2013). These findings indicate that both consecutive‐task and dual‐task interventions can modulate aversive memories shortly after encoding or retrieval.

A key distinction between the two interventions is that, in dual‐task interventions, the memory hotspot is deliberately recalled *while* simultaneously performing a secondary task. In contrast, consecutive‐task interventions use a brief reminder cue *before* the intervention to orient and reactivate the memory (e.g., Holmes et al., 2010; James et al., 2015). Both approaches are grounded in cognitive science and involve memory activation or reactivation and imagery‐interfering tasks that may modulate the emotional memory, though the necessity and degree of memory activation/reactivation may differ. One study directly compared the two approaches but used a Tetris‐like intervention that deviated from standard Tetris and typical procedures (Asselbergs et al., 2018). The Tetris‐like game in this study used blocks of three squares rather than Tetris’ four‐square blocks; required forming two‐by‐two squares rather than lines; and likely involved less mental rotation, making it an easier task. Thus, it remains unclear whether these findings generalize to interventions using standard Tetris gameplay.

The current study directly compared the effectiveness of the two interventions designed to target intrusive memories, which were induced using the trauma film paradigm (Holmes & Bourne, 2008). Participants were first shown an aversive film and were then assigned to one of three groups: (a) Tetris without memory recall task, (b) Tetris with memory recall task, or (c) no‐task control. We used original Tetris gameplay as an intervention because it has been used in research on both consecutive‐task (Holmes et al., 2009) and dual‐task (Engelhard et al., 2010) interventions. Before and after each intervention, participants rated the vividness and unpleasantness of their worst memory of the film. During the following week, they recorded film intrusions in a diary. Despite the procedural differences between the interventions, we expected that both Tetris conditions would reduce intrusive memories, as well as memory vividness and unpleasantness, compared with the control condition.

### Method

#### Participants

All participants gave written informed consent and were compensated after the study with course credits or money (€24). The final sample (after exclusion criteria were applied) consisted of 90 Dutch‐speaking students (*n* = 64 women, *n* = 26 men, *M*_age_ = 20.83 years, range: 17–28 years) who were evenly and randomly assigned to each of three groups. The local ethics committee approved this study, with data collection occurring from November 2016 to February 2017.

#### Procedure

Participants were tested individually in a large soundproof room without windows. On Day 1, participants provided informed consent and completed baseline measures: Beck Depression Inventory–II (BDI‐II; Beck et al., 1996), State Trait Anxiety Inventory–Trait (STAI‐T; Spielberger et al., 1983), and Traumatic Experiences Questionnaire (TEQ; Foa, 1996; see Supplementary Materials). After a 3‐min Tetris practice, they rated six prefilm mood scales (sadness, happiness, horror, fear, anxiety, and depression; 0 = *not at all*, 100 = *extremely*). A composite score was calculated to determine overall mood, with happiness reverse‐scored (Holmes et al., 2010). Participants then watched the trauma film and were instructed to view the events as a bystander, while the experimenter waited outside until the film finished. Postfilm, participants again rated mood and reported distress, attention to the film (0 = *not at all*, 100 = *extremely*), and prior film familiarity (“yes” or “no”). The trauma film consisted of a 9‐min, 24 s excerpt from “Irréversible” (Noé, 2002). The film follows two men, Marcus and Pierre, into a dark nightclub, where Marcus seeks out another man, leading to a violent confrontation that ends with Pierre killing the man by using a fire extinguisher to defend Marcus. In a validation study, this fragment elicited significant distress and intrusions (Arnaudova & Hagenaars, 2017). Participants watched the film on a 23‐in computer screen in a large, darkened room using over‐the‐ear headphones set to 50% volume.

Participants then completed a standardized 10‐min music filler task, rating the pleasantness of 15 fragments of classical music (Holmes et al., 2010; James et al., 2015). Immediately after the filler task, they selected the most distressing mental image from the film (i.e., the hotspot) and assigned a label to it. Directly after image selection, each participant was block‐randomized to one of the three conditions (i.e., Tetris without memory recall task, Tetris with memory recall task, or no‐task control). Before the intervention, participants were cued with the label and instructed to recall the hotspot and then rate its vividness and unpleasantness (0 = *not vivid/unpleasant*, 100 = *very vivid/unpleasant*).

In the Tetris without memory recall task condition, participants played Tetris consecutively for 10 min (e.g., Holmes et al., 2009). In the Tetris with memory recall task condition, participants were asked to recall the memory and simultaneously played Tetris for 25 intervals of 24 s, separated by 10 s breaks during which they were reminded to recall the memory while playing Tetris (“Recall [label] and hold onto to it”; Engelhard et al., 2010). Total Tetris gameplay time was identical in the two active conditions. In the no‐task control condition, participants were given a short 10‐min break, during which they were instructed to remain seated and quiet and could think about anything without restrictions (e.g., Holmes et al., 2009; James et al., 2015). After each intervention, participants recalled the hotspot and rated its vividness and unpleasantness. The task was presented in E‐Prime 2.0 (Psychology Software Tools, Pittsburgh, PA).

Participants in the Tetris conditions played a commercial version of Tetris (Version 1.6.00, Electronic Arts, Inc.) on a 9.7‐in iPad, without sound or advertisement interruptions. In the game, differently colored four‐square blocks (i.e., *tetrominoes*) fall from the top of the screen and are moved, rotated, or dropped via touch controls to complete horizontal lines that then disappear and yield points. All participants started at Level 1, and block speed increased as more lines were cleared (Marathon mode); in the event of a “game‐over” (i.e., the lines reach the top of the screen and blocks have no room to fall), play resumes at Level 1. As in previous experiments (e.g., Holmes et al., 2009; James et al., 2015), participants were instructed to focus on the three upcoming tetrominoes displayed at the upper right of the screen. Participants were encouraged to mentally rotate and plan block placement, with visual rotation promoted by disabling the ghost piece.

After rating vividness and unpleasantness, participants rated task compliance, task difficulty, and task pleasantness (0 = *not at all*, 100 = *extremely;* see Supplementary Materials; Emily A. Holmes, personal communication, June 29, 2016). Participants also rated how much they recalled the memory hotspot *during* the intervention (0 = *not at all*, 100 = *always*). Directly after completing these ratings, participants were instructed to record the number of intrusive memories in a paper‐and‐pen diary over the following week. Before leaving the lab, they received a brief checklist containing six yes/no statements to ensure correct diary use, with instructions reiterated and clarified as needed (Emily A. Holmes, personal communication, 29 April 2016).

On Day 8, participants visited the lab and completed the diary compliance scale (0 = *not at all accurately*, 100 = *extremely accurately*), the Impact of Event Scale (IES; Horowitz et al., 1979), the PTSD Checklist for *DSM‐5* (PCL‐5; Weathers et al., 2013), an intrusion provocation task (see Supplementary Materials for more information), and handed in their diaries. The experimenter and participant together checked that all sections were completed and that the recorded intrusions were film‐related; the first author confirmed this independently after data collection. Participants then received an extensive debriefing, including a clip showing how the most aversive scenes were created using latex dolls and visual effects, similar to a procedure used by Kindt et al. (2008). Finally, participants were reimbursed for their time. The Ethical Committee of the Faculty of Social and Behavioral Sciences at Utrecht University (FETC15‐104) approved the experiments.

#### Measures

##### Intrusion diary

The experimenter explained the concept of an “intrusion,” defined as spontaneous mental images (e.g., pictures in the mind's eye), verbal thoughts (words or phrases), or a combination, to each participant (e.g., James et al., 2015). Participants recorded any film‐related intrusions in a pen‐and‐paper diary for 1 week. Intrusions were to be noted as soon as possible in the relevant time period (morning, afternoon, evening/night) or completed once daily if immediate recording was not possible. For each intrusion, participants rated distress, vividness, unpleasantness, arousal, and suddenness on a scale of 0 (*not at all*) to 10 (*extremely*). Only intrusions with image‐based content, including mixed image‐thought intrusions (e.g., Holmes et al., 2009), were analyzed.

##### Trauma‐related psychological distress

The 15‐item IES (Horowitz et al., 1979) measures psychological stress reactions after a major life event. Items are rated on a 4‐point scale (0 = *not at all*, 1 = *rarely*, 3 = *sometimes*, and 5 = *often*) with higher scores indicating higher levels of distress over the past 7 days. Seven items assess intrusions, and eight items assess avoidance. Participants were asked to rate items with respect to the film. The IES has demonstrated good validity, and the Intrusion and Avoidance subscales have typically shown high reliability (e.g., Cronbach's αs = .86 and .82, respectively; Sundin & Horowitz, 2003). In our sample, only the Intrusion subscale was analyzed, and it demonstrated adequate reliability, Cronbach's α = .71.

##### PTSD intrusion symptoms

The PCL‐5 (Weathers et al., 2013) is a 20‐item self‐report measure aligned with the PTSD symptom criteria outlined in the *Diagnostic and Statistical Manual of Mental Disorders* (5th ed.; *DSM‐5*; APA, 2013). For this study, only the five intrusion items were used; these items were adapted to assess past‐week film‐related symptoms. The PCL‐5 has demonstrated good convergent and discriminant validity, with excellent total‐scale reliability (Cronbach's α = .94); subscale reliability is generally not reported (Blevins et al., 2015). In our sample, reliability for the adapted Intrusion (Cluster B) subscale was borderline acceptable, Cronbach's α = .63.

#### Data analysis

JASP (Version 0.95.4) was used for null hypothesis significance test (NHST) and Bayesian hypothesis test (BHT) analyses. Bayes factors (BF), with default priors, are reported to aid interpretation of nonsignificant or expected equivalence results. We report BF_01_ (evidence for H_0_ over H_1_) for comparisons between two groups. For factorial designs, we report BF_excl_ (matched across models), which reflects the evidence for excluding a specific predictor or interaction from the model. A BF_excl_ of 10 suggests that the data are 10 times more likely under models that exclude the predictor or interaction than those that include it.

For most NHST and BHT analyses, analyses of variance (ANOVAs) were conducted with group (three levels) as a between‐subjects factor and time (two levels) included where applicable. Assumptions were checked for all analyses (see Supplementary Materials), and corrections were applied when violations were detected. Planned contrasts were used to further examine significant main effects and interactions. Four participants had a single missing value (three for the IES, one for the TEQ). These missing values were handled using mean imputation.

### Results

#### Film‐related mood, distress, and attention

The film effectively increased negative mood, *F*(1, 87) = 437.87, *p* < .001, η^2^_p_ = .83, with no group interaction, *F*(2, 87) = 0.96, *p* = .286, η^2^_p_ = .02, BF_excl_ = 4.10. Participants found the film distressing (*M* = 78.42, *SD* = 17.49) and reported a high level of attention to the film (*M* = 90.71, *SD* = 8.96), with no group differences, *F*(2, 87) = 0.17, *p* = .844, η^2^_p_ < .01, BF_01_ = 8.78, and *F*(2, 87) = 0.78, *p* = .462, η^2^_p_ = .02, BF_01_ = 5.43, respectively. For all descriptive statistics, see Supplementary Table S1.

#### Memory vividness and unpleasantness

For memory vividness, there was a main effect of time, *F*(1, 87) = 93.56, *p* < .001, η^2^_p_ = .52, but not group, *F*(2, 87) = 0.19, *p* = .824, η^2^_p_ < .01, BF_01_ = 4.84. However, there was a Group x Time interaction, *F*(2, 87) = 4.26, *p* = .017, η^2^_p_ = .09. Follow‐up contrasts for the interaction on the pretest–posttest difference scores refer to multiplicity‐corrected post hoc tests, as JASP does not support follow‐up Bayesian contrasts. These results revealed that there was no difference between the Tetris without memory recall task and the no‐task control conditions, *t*(87) = 0.39, *p* = .694, *d* = 0.10, BF_01_ = 3.42. Vividness decreased more in the Tetris with memory recall task condition than in the no‐task control condition, *t*(87) = 2.31, *p* = .023, *d* = 0.60, and the Tetris without memory recall task condition, *t*(87) = 2.70, *p* = .008, *d* = 0.70 (see Figure 1, Panel A).

**FIGURE 1:**
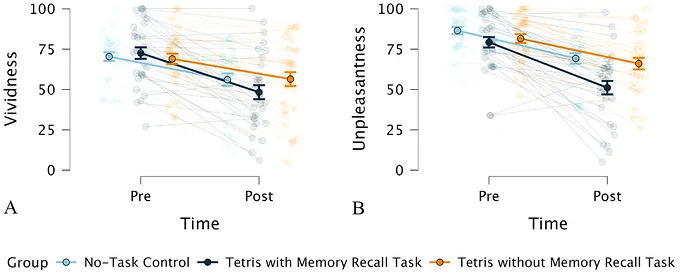
Memory vividness and memory unpleasantness per experimental group *Note*: Figure 1A refers to vividness, Figure 1B refers to unpleasantness. Individual data points are shown as lines connecting each participant's pretest (Pre) and posttest (Post) scores. Group means appear as larger, darker dots with standard error bars.

For memory unpleasantness, there was again a main effect of time, *F*(1, 87) = 116.29, *p* < .001, η^2^_p_ = .57, and an effect of group, *F*(2, 87) = 5.252, *p* = .007, η^2^_p_ = .11. However, these effects may be better explained by the Group x Time interaction, *F*(2, 87) = 4.39, *p* = .015, η^2^_p_ = .09. We compared group difference scores, which again showed there was no difference between the Tetris without memory recall task condition and the no‐task control condition, *t*(87) = 0.37, *p* = .709, *d* = .10, BF_01_ = 3.42. Unpleasantness decreased more in the Tetris with memory recall task condition than in the no‐task control condition, *t*(87) = 2.36, *p* = .021, *d* = 0.61, and the Tetris without memory recall task condition, *t*(87) = 2.73, *p* = .008, *d* = 0.71 (see Figure 1, Panel B).

#### Memory recall during the intervention

There was strong evidence of differences in memory recall, *F*(2, 54.53) 106.80, *p* < .001, η^2^_p_ = .61. Post hoc comparisons indicated that all groups differed from each other, *t*s(87) = 5.67–11.64, *p*s < .001, *d*s = 1.46–3.01, with memory recall highest in the Tetris with memory recall task condition (*M* = 74.55 *SD* = 18.53), followed by the no‐task control condition (*M* = 44.63, *SD* = 26.84), and lowest in the Tetris without memory recall task condition (*M* = 13.13, *SD* = 13.72). These findings confirm that the manipulation of deliberate, simultaneous, and continuous memory recall was effective.

#### Intrusive memories

All participants handed in complete 1‐week intrusion diaries, reporting an average of 3.63 intrusions per week (*SD* = 3.63) and high diary compliance (*M* = 77.62, *SD* = 11.97). All intrusions were related to the film, with most involving the focal fire extinguisher assault scene. There were, however, no between‐group differences in intrusion frequency, *F*(2, 55.61) = 0.82, *p* = .448, η^2^_p_ = .02, BF_01_ = 5.57 (see Figure 2), or diary compliance, *F*(2, 87) = 1.39, *p* = .255, η^2^_p_ = .03, BF_01_ = 3.36.

**FIGURE 2:**
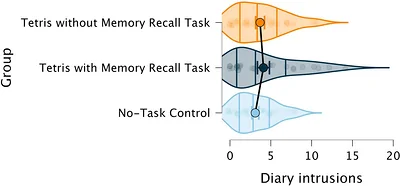
Diary intrusions per experimental group *Note*: Individual data points are shown as faint dots. Means appear as larger, darker dots, with standard error bars.

Participants also retrospectively rated film‐related intrusions over the past week, but no differences were observed in IES scores (Intrusion subscale), *F*(2, 87) = 0.13, *p* = .876, η^2^_p_ < .01, BF_01_ = 9.05, or PCL‐5 scores (Intrusions subscale), *F*(2, 87) = 1.27, *p* = .287, η^2^_p_ = .03, BF_01_ = 3.70.

## EXPERIMENT 2

Although the consecutive‐task and dual‐task interventions differ procedurally in how they engage memory, it remains unclear whether this reflects distinct underlying cognitive mechanisms. Specifically, the consecutive‐task procedure uses a brief reminder cue to reactivate the memory, whereas the dual‐task procedure involves continuous memory recall. These differing forms of memory engagement may place distinct demands on cognitive resources, which could influence the level of imagery interference and, consequently, the interventions’ effectiveness in reducing intrusive memories (e.g., Holmes et al., 2009; Mertens et al., 2021).

A consecutive‐task procedure, in which the reminder cue and Tetris are presented sequentially, may confer lower cognitive demands than a dual‐task procedure, where the two tasks compete for resources simultaneously. Consecutive‐task effects are thought to arise from Tetris interfering with excessive perceptual processing of the traumatic memory, selectively disrupting sensory components that drive intrusions. This assumes that the memory—although not deliberately recalled during the procedure—remains active in WM. In contrast, the dual‐task procedure requires direct engagement with the memory: It is deliberately recalled and, in turn, maintained in one's WM while they simultaneously perform a secondary task (e.g., playing Tetris).

To assess how brief memory reactivation (in the consecutive‐task procedure) versus sustained recall (in the dual‐task procedure) differentially affects cognitive load, a random interval repetition (RIR) task can be used. In an RIR task, participants respond as quickly as possible to randomly administered stimuli while simultaneously performing another cognitive activity. In the consecutive‐task condition, the memory is briefly reactivated prior to responding, whereas in the dual‐task condition, participants continuously keep the memory in mind throughout the RIR task (Vandierendonck et al., 1998). Slower reaction times relative to an RIR‐only baseline provide a sensitive and precise measure of cognitive load due to the large number of observations.

Reaction‐time slowing is a well‐established index of cognitive load for secondary tasks, such as playing Tetris (Engelhard et al., 2010), “Tetris‐like” visuospatial games (Asselbergs et al., 2018), horizontal eye movements (van den Hout et al., 2011), and counting (Engelhard et al., 2011; van Schie et al., 2019), reliably impairing response speed. Importantly, cognitive load is also increased by the deliberate recall of an emotional memory itself (van Veen et al., 2016). Therefore, we expected that the dual‐task procedure (i.e., RIR with memory recall), which involves active memory recall while performing the RIR task, would increase reaction times compared to the RIR‐only condition. We also expected increased reaction times in the consecutive‐task procedure (i.e., RIR without memory recall) compared to RIR‐only. Whether slowing would be greater in the dual‐task condition than in the consecutive‐task condition, given differences in memory engagement, remains uncertain and was examined.

### Method

#### Participants

Dutch‐speaking participants (*N* = 45, *n* = 33 women, *n* = 12 men; *M*_age_ = 21.20 years, range: 18–27 years) performed a four‐blocked RIR task (van den Hout et al., 2011) and were equally assigned to one of three conditions: (a) RIR without memory recall task, (b) RIR with memory recall task, or (c) RIR only.

#### Procedure

All participants received a short practice RIR task consisting of 24 white circles. Next, participants in the RIR without and with memory recall task groups, but not the RIR‐only group, watched the trauma film, selected a hotspot, and rated its unpleasantness and vividness, following the pretest procedure for Experiment 1. Next, all three groups performed the RIR task, with only participants in the RIR with memory recall task condition instructed to simultaneously recall their hotspot.

For the RIR task, participants were instructed to press a button with their dominant‐hand index finger when they saw a 3‐cm diameter white circle appear on a black background. Circles were presented for 500 ms, followed by an interstimulus interval of 400 or 1000 ms. Trial duration selection was quasirandom, with maximally four identical intervals in a row. Reaction times were measured from circle onset. A total of 148 circles were presented equally over four blocks to examine early and late effects on WM load. Afterward, participants in both the RIR with and without memory recall task groups, but not the RIR‐only group, rated the film‐ and task‐related questions.

#### Measures

Participants in the RIR with and without memory recall task groups, but not the RIR‐only group, completed film‐ and task‐related measures. They were asked to rate the degree of their attention to the film (0 = *not at all*, 100 = *extremely*), their familiarity with the film (“yes” or “no”), and how much the hotspot was in their mind during the RIR task (0 = *never*, 100 = *always*). Results for attention and familiarity are reported in the Supplementary Materials.

### Results

#### Reaction time

Mean values for outlier correction reaction times below 200 ms or above 1500 ms were excluded from the analyses (Cuperus et al., 2016). This occurred, on average, in 20.24 of 148 trials (13.5%) with no group differences, *F*(2, 42) = 0.60, *p* = .552, η^2^_p_ = .03, BF_01_ = 4.00.

A Block x Condition ANOVA on reaction times showed an interaction effect, *F*(4.95, 104.01) = 2.56, *p* = .032, η^2^_p_ = .11, explaining the main effect of condition, *F*(2, 42) = 3.23, *p* = .050, η^2^_p_ = .13. There was no main effect of block, *F*(2.48, 104.01) = 1.64, *p* = .192, η^2^_p_ = .04, BF_01_ = 5.70. The interaction (see Figure 3) was followed by three contrasts comparing the groups within each block. In Block 1, the groups differed from each other, *F*(2, 26.13) = 4.51, *p* = .021, η^2^_p_ = .26. Importantly, participants in the RIR with memory recall task group showed increased reaction times compared to the other two groups, *t*s(42) = 3.18–3.41, *p*s = .001–.003, *d*s = 1.16–1.25, whereas the reaction times for the RIR without memory recall task and RIR only groups were comparable, *t*(42) = 0.24, *p* = .814, *d* = 0.09, BF_01_ = 2.76. The dual‐task effect was transient and disappeared in the last three blocks, *F*s = 0.71–2.03, *p*s = .153–.500, η_p_^2^ = .03–.10 with Bayes factors remaining somewhat stable over blocks for Block 2, BF_01_ = 1.35; Block 3, BF_01_ = 4.13; and Block 4, BF_01_ = 2.73). For all descriptive statistics, see Supplementary Table S2.

**FIGURE 3:**
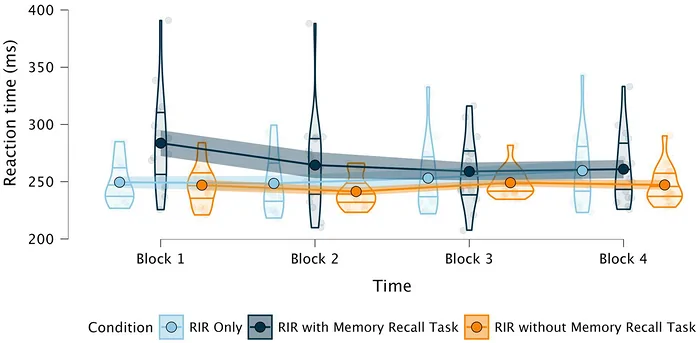
Reaction times per condition for the random interval repetition task *Note*: Violin plots with individual data points represented by faint dots; group means are larger blue, black, and orange dots, with a standard error ribbon.

#### Memory recall during the RIR task

As expected, participants in the RIR with Memory Recall Task group reported higher memory recall (*M* = 70.80, *SD* = 9.53) than those in the RIR without memory recall task group (*M* = 54.93, *SD* = 24.92), *t*(18.01), *p* = .033, *d* = 0.84.

## DISCUSSION

We examined whether two procedurally distinct Tetris interventions could reduce intrusive memories of analogue trauma and self‐reported vividness and unpleasantness of a memory hotspot, compared to a control condition. The two interventions differed only in the degree of deliberate memory recall. Experiment 1 showed comparable numbers of intrusive memories across the Tetris and control conditions. Decreases in memory vividness and unpleasantness were largest among participants in the Tetris with memory recall task condition compared to the other two conditions. Experiment 2 revealed that only deliberate recall (central to the dual‐task intervention) objectively taxed WM.

Consistent with the literature (e.g., Engelhard et al., 2010; Mertens et al., 2021), a dual‐task Tetris intervention reduced both memory vividness and unpleasantness more than a no‐intervention control condition. In contrast, the consecutive Tetris intervention did not reduce vividness or unpleasantness relative to the control condition, consistent with findings that secondary tasks reduce these measures only when performed simultaneously with memory recall, but not without recall (Gunter & Bodner, 2008, though see van Schie & van Veen, 2023). However, our results on intrusive memories diverge from earlier trauma film studies showing that playing Tetris after a reminder cue reduced the number of intrusions compared to a no‐task control condition (e.g., Hagenaars et al., 2017; Holmes et al., 2009, 2010). Although a 2023 meta‐analysis reported overall reductions in intrusions following visuospatial interventions like Tetris, all included studies had at least some risk of bias, and four studies had a high risk of bias (Asselbergs et al., 2023). In contrast, a large multilab replication (Wessel et al., 2025) and several recent studies failed or partially failed to detect this effect (Broughill et al., 2025; Hemi et al., 2023; Kehyayan et al., 2024; Matura et al., 2025), though the null effect reported by Kehyayan et al. (2024) may reflect their crossover design. Null findings are often attributed to excessive methodological deviation, whereas positive results under similar deviations are seen as robust, complicating interpretation (Button et al., 2016). Nevertheless, examining methodological or procedural deviations remains important for understanding failures to observe an effect of Tetris. Clarifying these boundary conditions is also important for informing safe and effective use in clinical practice.

One possible reason for the lack of effects for the Tetris interventions may be the film used. Although the film we used was validated (Arnaudova & Hagenaars, 2017), it elicited fewer intrusions compared to other studies using trauma films (e.g., Holmes et al., 2009, 2010). Unlike those films—typically composed of multiple short accident clips (e.g., Hagenaars et al., 2017; James et al., 2015)—our film was a single continuous clip with a different theme (i.e., murder). Moreover, low intrusion frequencies have been reported without limiting intervention effects (e.g., Krans et al., 2009; Stuart et al., 2006), including in Tetris studies (Hagenaars et al., 2017). Together, this suggests that the absence of an effect is unlikely to reflect a film‐induced floor effect.

Another explanation concerns the active selection of the memory hotspot and the preintervention ratings of unpleasantness and vividness. In previous trauma film studies using a Tetris‐based intervention, brief standardized visual cues from the film have typically been used to reactivate the memory (e.g., James et al., 2015). In contrast, preintervention ratings may act as a stronger reminder cue, prompting participants to reengage with the memory more fully. Such retrieval could engage exposure‐like processes, thereby activating mechanisms that reduce later intrusions. Critically, if this is the case, the control condition should also show fewer intrusions, reducing between‐condition differences. However, if a stronger reminder cue had indeed facilitated memory exposure, it should also have imposed a cognitive load in the RIR without memory recall task condition, which it did not. Experiment 2 showed that only deliberate memory recall, as implemented in the dual‐task procedure, and not a preintervention rating alone (i.e., without deliberate memory recall), produced cognitive load.

The findings of Experiment 2 align with previous research showing that additional cognitive tasks, such as memory recall, impose a WM load (van den Hout et al., 2014; van Veen et al., 2016). However, this effect was transient, suggesting that the initial impact of memory recall on cognitive resources quickly diminished. This is consistent with the rapid decay of WM traces when attention is diverted (Barrouillet et al., 2004), particularly because memory recall instructions were only given once at the start of the task and were not repeated during the RIR task for the RIR with memory recall group. Interestingly, the RIR without memory recall and the RIR‐only conditions showed no differences in reaction times across blocks, suggesting that the memory recall component specifically drove the initial increase in reaction times. This contrasts with the presumed mechanism of the consecutive‐task procedure, which assumes the aversive memory remains in WM even without deliberate recall and can be disrupted by a secondary task (e.g., playing Tetris). Overall, these results show that the cognitive load of memory activation is larger in the dual‐task procedure compared to the consecutive‐task procedure.

The findings from both Experiments 1 and 2 raise the question: Which components of the Tetris intervention are responsible for modulating intrusive memories? Identifying these critical elements may help elucidate the cognitive mechanisms underlying the intervention, which is particularly relevant given its application in clinical settings and potential for safe and optimized use. Some researchers have suggested that, specifically, the engagement of visuospatial processing (e.g., mental rotation, image formation) in Tetris prevents the processing of visual images of a traumatic event (e.g., Holmes et al., 2009, 2010; Stuart et al., 2006). However, others argue that general taxation, with or without memory recall, and not visuospatial taxation, is necessary for intrusion modulation (e.g., Gunter & Bodner, 2008; Pearson & Sawyer, 2011; van den Hout & Engelhard, 2012). Indeed, studies have found intrusion‐reducing effects for nonvisuospatial tasks (Asselbergs et al., 2023; Hagenaars et al., 2017; Kessler et al., 2020).

One proposed cognitive mechanism is WM taxation, which may underlie the effects of active Tetris‐based interventions like those used in Experiment 1. Playing Tetris is known to tax WM (e.g., Asselbergs et al., 2018; Engelhard et al., 2010), and deliberate memory recall also imposes WM demands (van den Hout et al., 2014; van Veen et al., 2016). However, it remains unclear whether a brief reminder cue, such as the preintervention memory rating, similarly taxes WM. In Experiment 2, such a cue did not produce measurable cognitive load, whereas deliberate memory recall as part of the dual‐task procedure increased WM load. This finding challenges the presumed mechanism of the consecutive‐task procedure, which posits that the aversive memory remains in WM even in the absence of explicit retrieval, allowing a secondary task (e.g., playing Tetris) to interfere with its sensory representation. This, therefore, raises the question of whether a memory that does not measurably tax WM can still be disrupted via WM‐based interference.

These results also have implications for the necessity of brief reminder cues used in trauma film studies when the film and intervention occur on the same day. Although interventions combining a reminder cue with a visuospatial task have typically been compared to either a no‐task control condition (Asselbergs et al., 2023) or a reminder‐only condition (Lau‐Zhu et al., 2021), they have not been directly contrasted with a no‐reminder condition. Such a comparison would help to clarify whether a reminder cue is required when the analogue trauma is recent. James et al. (2015) suggested that when the trauma film is shown a day earlier and Tetris is played a day later, a reminder cue preceding Tetris gameplay is necessary. In same‐day designs, however, the memory is likely still active, making the necessity of a reminder cue an open empirical question.

This research has potential limitations. First, the sex distribution across conditions in both experiments was somewhat unbalanced, which may have influenced intrusive reexperiencing in Experiment 1, given the higher prevalence of these symptoms in women (Olff, 2017), as well as reaction times, because women tend to show slower and more variable responses (Der & Deary, 2006). Second, in Experiment 2, memory recall instructions were provided only once at the beginning of the task and were not repeated during the memory recall task, which may have reduced the overall WM load because of a rapid decay of WM traces. At the same time, the laboratory setting allowed for precise control and measurement, supporting investigation of the mechanisms underlying intrusive memories. Building on these strengths, future laboratory and clinical research should further examine why visuospatial interventions such as Tetris are sometimes effective in reducing intrusions and sometimes not. In particular, the conditions under which effects emerge, and why similar effects can also arise from nonvisuospatial tasks, warrant further investigation.

In summary, Experiment 1 demonstrated that combining Tetris with a memory recall task produced the largest reductions in memory vividness and unpleasantness, but did not affect intrusion frequency. Experiment 2 extended these findings by showing that only deliberate, simultaneous recall imposed a measurable WM load. Overall, these results suggest that a dual‐task intervention involving Tetris can selectively modulate how the memory hotspot is experienced, with the deliberate memory recall component specifically taxing WM. However, it remains unclear which methodological factors (e.g., timing of recall, task features, WM demands) account for the inconsistent effects of Tetris on intrusive memories. Future research should therefore focus on the critical elements of Tetris‐based interventions. Clarifying these mechanisms is essential for advancing theoretical understanding while informing safe and effective clinical applications.

## AUTHOR NOTE

This research was supported with a TOP grant (40‐00812‐98‐12030) from the Netherlands Organization for Health Research and Development, awarded to Marcel van den Hout and Iris Engelhard. **Kevin van Schie** worked on this project under this TOP grant. Iris Engelhard was supported by a Vici grant (453‐15‐005) from the Netherlands Organization for Scientific Research.

We wish to thank Johan Verwoerd and Ineke Wessel for providing the trauma film, Sarah Goerres and Derk Wisman for their assistance with Experiment 1, Rinske Heuvel for her help with Experiment 2, and Muriel Hagenaars for valuable suggestions regarding the study design and discussion. The authors gratefully acknowledge Marcel A van den Hout for his important role in the conception and design of the study and for drafting the initial version of the manuscript. Prof. van den Hout declined authorship of the current revision due to his retirement.

## OPEN PRACTICES STATEMENT

Neither of the studies reported in this article was formally preregistered. Annotated JASP files and supplementary information (e.g., additional analyses) are publicly available on the Open Science Framework: https://osf.io/4aruq/

Requests for the materials can be sent via email to the lead author at k.vanschie@tilburguniversity.edu

## AUTHOR CONTRIBUTIONS

**Kevin van Schie**: conceptualization, methodology, software, formal analysis, investigation, data curation, writing—original draft, writing—review and editing, visualization, supervision, project administration. **Henrik Kessler**: methodology, writing—review and editing. **Iris M. Engelhard**: conceptualization, methodology, resources, writing—review and editing, supervision.

## Supporting information



SUPPORTING INFORMATION
