# Supplementary material for: Effects on intrusive memories of playing Tetris with or without simultaneous and deliberate memory recall
Source: J Trauma Stress. 2026 Jul 1;39(5):897–908. doi: 10.1002/jts.70104 (PMC13639210; doi:10.1002/jts.70104)
Supplement: Supplementary file 1 [file JTS-39-897-s001.docx]

**Experiment 1**

**Method**

***Participants screening, exclusion and power***

Individuals were excluded from participation if (a) they did not give informed consent (*n* = 1), (b) reported knowledge about the working mechanism of EMDR therapy (*n* = 5), (c) had participated in previous EMDR or trauma film experiments (*n* = 3), (d) used medication that affected memory or concentration, (e) had been diagnosed with a psychiatric disorder, (f) having been the victim or witness of extreme physical violence, and (g) were prone to fainting when seeing blood. The latter criteria (c through g) were assessed with self-reports. There were two data exclusions: one participant showed non-compliant behavior (talking constantly to the experimenter while playing Tetris); another participant did not show up on Day 8.

Original studies using the Tetris intervention reported very large effect sizes compared to no-task control groups (Holmes et al., 2009, 2010). A power analysis in G*Power with 80% power, an effect size of *d* = 0.8, and α = 0.05 indicates a required total sample size of 42. A post hoc sensitivity analysis (80% power, α = 0.05) shows that between group comparisons (with *n* = 30) were able to detect effect sizes as small as *d* = 0.65.

***Tasks***

**Trauma film.** The film depicts a coherent narrative of two men (Marcus & Pierre) entering a dark and sordid nightclub. From the start, Marcus is impatiently looking for another man. Towards the end of the film Marcus finds that person and the two get into a violent fight. Marcus falls to the ground and the other man breaks Marcus’ arm. Pierre defends Marcus by using a fire extinguisher to crush the other man’s skull. The film ends after this brutal murder. The film is in French (without subtitles) and is accompanied by the original soundtrack of continuous low frequency horn sounds (Nixon et al., 2009; Weidmann et al., 2009; Wessel et al., 2010).

***Baseline Measures***

**Beck-depression inventory-II (BDI-II).** The BDI-II (Beck et al., 1996) is a 21-item validated self-report questionnaire that assesses depressive mood. Items measure different symptoms that are related to depression. All items are scored on a scale ranging from 0 to 3, and higher sum total scores (ranging from 0 to 63) indicate higher severity of depressive mood. The BDI-II generally shows good reliability in psychiatric populations (α = .91). In the current study (with a college student population) the reliability was sufficient (α = .73)

**State trait anxiety inventory – trait (STAI-T).** The STAI-T measures trait anxiety and consists of 20 items, which are scored on a four-point scale from 1 (*almost never*) to 4 (*almost always*). (Spielberger et al., 1983). A number of items need to be reverse scored. Sum scores on the STAI-T range from 20 to 80 with higher scores representing higher levels of anxiety. Overall, the STAI-T shows good reliability in different populations (α ≥ .89), as well as in the current study (α = .83). Regarding its validity, the STAI‑T has been recently recognized as primarily reflecting negative affectivity rather than anxiety per se (Knowles & Olatunji, 2020).

**Traumatic experiences questionnaire (TEQ).** The TEQ is a 12-item checklist adapted from the Criterion A list of the Posttraumatic Diagnostic Scale (Foa, 1995) as per previous studies (e.g., Holmes et al., 2010). For a list of 12 traumatic events participants indicated whether or not they experienced or witnessed the event (e.g., a serious accident, life-threatening illness). Total scores ranged from 0 (*no traumatic event experienced or witnessed*) to 12.

***Measures***

**Intrusion-provocation task (IPT).** The IPT was adapted from James et al. (2015) and was included as an exploratory measure. To construct the IPT, nine static film images were blurred (Gaussian Blur set 20.0 in GIMP2.8) and shown on a computer for 2s each in chronological order. Participants then closed their eyes for 2 min and pressed a designated key on a keyboard whenever a film-related intrusion occurred.

**Results**

***Assumption checks***

Outliers were identified within each group using the interquartile range (IQR) criterion (values falling outside Q1 − 1.5×IQR or Q3 + 1.5×IQR) and were winsorized to the corresponding IQR boundary. Homogeneity of variances was assessed using Levene’s test of equality of variances; when this assumption was violated, Welch’s ANOVA was used. Normality was assessed by inspecting the distributions. Given equal group sizes (*n* = 30), ANOVA is robust to moderate deviations from normality according to the Central Limit Theorem. Other assumptions (independence of observations, a continuous dependent variable, and a categorical independent variable) were met by design. Because the within-subject factor Time contained only two levels (pre, post), the assumption of sphericity was inherently satisfied.

***Baseline measures***

Overall, the three groups showed equivalence for Age, *F*(2, 87) = 0.37, *p* = .695, n^2^_p_ < .01, BF_01_ = 7.53; BDI-II scores, *F*(2, 56.37) = 1.33, *p* = .273, n^2^_p_ = .03, BF_01_ = 3.24; and reported exposure to traumatic events, *F*(2, 87) = 0.02, *p* = .985, n^2^_p_ < .01, BF_01_ = 9.93 (see Table S1). There was insufficient evidence for trait anxiety to draw meaningful conclusions, *F*(2, 55.30) = 2.45, *p* = .095, n^2^_p_ = .06, BF_01_ = 1.06 (also see Table S1)

***Task performance***

Task compliance, task pleasantness, and task difficulty were compared (see Table S1). There were differences between conditions in task compliance, *F*(2, 54.93) = 6.78, *p* = .002, n^2^_p_ = .12. Tukey-corrected post hoc test showed No-Task Control reported higher compliance compared to Tetris with Memory Recall Task, *t*(87) = 3.51, *p* = .002, *d* = .91, while the other two comparisons were not significant, *t*s(87) ≤ 1.89, *p*s ≥ .149, *d*s ≤ .42, BFs_01_ ≤ 1.59

For task pleasantness, there were overall differences, *F*(2, 55.24) = 14.22, *p* < .001, n^2^_p_ = .22. Post hoc tests suggested that participants found the Tetris without Memory Recall Task more enjoyable than the No-Task Control, *t*(87) = 4.90, *p* < .001, *d* = 1.27, and Tetris with Memory Recall Task, *t*(87) = 2.45, *p* = .042, *d* = .63. The Tetris with Memory Recall Task appeared to be more enjoyable than the No-Task Control, *t*(87) = 2.50, *p* = .043, *d* = .63.

There were differences between conditions in task difficulty, *F*(2, 55.90) = 7.29, *p* = .002, n^2^_p_ = .12. Post-hoc follow-up showed that both Tetris without Memory Recall Task and Tetris with Memory Recall Task were experienced as more difficult than No-Task Control, *t*s(87) ≥ 2.48, *p*s ≤ .040, *d*s ≥ 0.64. However, there was no difference between the Tetris conditions with and without memory recall in task difficulty, *t*(87) = 0.86, *p* = .664, *d* = .22, BF_01_ = 2.94.

***Intrusive memories***

There were no differences on Intrusion Provocation Task performance between the group, *F*(2, 87) = 0.09, *p* = .912, n^2^_p_ < .01, BF_01_ = 9.34. Participants who reported at least one intrusion in their diary (*n* = 77) were included in the analyses of diary intrusion characteristics (distress, vividness, unpleasantness, arousal, and suddenness), and for participants who reported multiple intrusions, averaged ratings for each characteristic were used (see Table S1). There were however no significant differences between groups for any of the intrusion characteristics, *F*s(2, 74) ≤ 1.73, *p*s ≥ .185, n^2^_p_ ≤ .05, BFs_01_ ≥ 2.36.

**Experiment 2**

**Results**

***Assumption checks***

Assumptions were checked following the same general procedure as in Experiment 1. Because the within-subject factor Time contained four levels (Blocks 1–4), violations of sphericity were addressed using the Greenhouse–Geisser correction.

***Film and task related measures***

There were differences in attention to the film: the RIR task without Memory Recall Task group reported more attention compared to the RIR task with Memory Recall Task group, *t*(28), *p* = .004, *d* = 1.14 (also see Table S2). Two participants in the RIR without Memory Recall Task condition reported familiarity with the film compared to none in the other condition.

**References**

Beck, A. T., Steer, R. A., & Brown, G. K. (1996). *Beck Depression Inventory-II (BDI-II)*. The Psychological Corporation, Harcourt Brace.

Foa, E. B. (1995). *The Posttraumatic Diagnostic Scale (PDS) Manual*. National Computer Systems.

Holmes, E. A., James, E. L., Coode-Bate, T., & Deeprose, C. (2009). Can Playing the Computer Game “Tetris” Reduce the Build-Up of Flashbacks for Trauma? A Proposal from Cognitive Science. *PLoS ONE*, *4*(1), e4153. https://doi.org/10.1371/journal.pone.0004153

Holmes, E. A., James, E. L., Kilford, E. J., & Deeprose, C. (2010). Key Steps in Developing a Cognitive Vaccine against Traumatic Flashbacks: Visuospatial Tetris versus Verbal Pub Quiz. *PLoS ONE*, *5*(11), e13706. https://doi.org/10.1371/journal.pone.0013706

James, E. L., Bonsall, M. B., Hoppitt, L., Tunbridge, E. M., Geddes, J. R., Milton, A. L., & Holmes, E. A. (2015). Computer game play reduces intrusive memories of experimental trauma via reconsolidation-update mechanisms. *Psychological Science*, *26*(8), 1201–1215. APA PsycInfo (2015-36637-005). https://doi.org/10.1177/0956797615583071

Knowles, K. A., & Olatunji, B. O. (2020). Specificity of trait anxiety in anxiety and depression: Meta-analysis of the State-Trait Anxiety Inventory. *Clinical Psychology Review*, *82*, 101928. https://doi.org/10.1016/j.cpr.2020.101928

Nixon, R. D. V., Cain, N., Nehmy, T., & Seymour, M. (2009). Does post-event cognitive load undermine thought suppression and increase intrusive memories after exposure to an analogue stressor? *Memory*, *17*(3), 245–255. https://doi.org/10.1080/09658210802592353

Spielberger, C. D., Gorsuch, R. L., Lushene, R. E., Vagg, P. R., & Jacobs, G. A. (1983). *Manual for the State-Trait Anxiety Inventory (Form Y)*. Consulting Psychologists Press.

Weidmann, A., Conradi, A., Gröger, K., Fehm, L., & Fydrich, T. (2009). Using stressful films to analyze risk factors for PTSD in analogue experimental studies – which film works best? *Anxiety, Stress & Coping*, *22*(5), 549–569. https://doi.org/10.1080/10615800802541986

Wessel, I., Huntjens, R. J. C., & Verwoerd, J. R. L. (2010). Cognitive control and suppression of memories of an emotional film. *Journal of Behavior Therapy and Experimental Psychiatry*, *41*(2), 83–89. https://doi.org/10.1016/j.jbtep.2009.10.005

**Table S1**

*Baseline, Film, and (Intrusive) Memory Measures for the Three Groups.*

|  | Tetris without Memory Recall Task | Tetris with Memory Recall Task | No-Task Control |
| --- | --- | --- | --- |
| Baseline |  |  |  |
| Sex: men/women (*n*) | 6 / 24 | 11 / 19 | 9 / 21 |
| Age (years) | 20.87 (2.46) | 21.10 (2.66) | 20.53 (2.62) |
| BDI-II | 6.67 (4.92) | 5.74 (3.40) | 4.97 (3.11) |
| STAI-T | 37.97 (9.60) | 34.93 (6.40) | 33.55 (5.21) |
| TEQ | 0.57 (0.85) | 0.57 (0.88) | 0.60 (0.85) |
| Film |  |  |  |
| Pre-film negative mood ^a^ | 15.04 (7.23) | 14.60 (8.17) | 13.84 (6.45) |
| Post-film negative mood ^a^ | 51.44 (17.28) | 53.47 (19.39) | 56.58 (16.60) |
| Distress (related to the film) | 78.76 (17.84) | 79.54 (17.65) | 76.85 (17.49) |
| Attention (to the film) | 89.13 (9.40) | 91.98 (8.86) | 91.00 (8.67) |
| Familiarity (with the film) (*n*) | 1 | 0 | 0 |
| Memory Vividness & Unpleasantness |  |  |  |
| Pre-intervention vividness | 69.02 (17.32) | 72.57 (19.66) | 70.38 (14.57) |
| Post-intervention vividness | 56.43 (23.61) | 48.33 (23.73) | 56.10 (21.13) |
| Pre-intervention unpleasantness | 81.57 (15.12) | 79.27 (17.56) | 86.53 (12.27) |
| Post-intervention unpleasantness | 66.03 (19.66) | 51.13 (22.86) | 69.28 (17.97) |
| Task performance |  |  |  |
| Task compliance | 88.00 (11.20) | 83.23 (14.11) | 93.53 (7.96) |
| Task pleasantness | 72.38 (15.70) | 58.58 (24.42) | 44.80 (24.11) |
| Task difficulty | 34.27 (25.05) | 39.53 (26.95) | 19.15 (17.81) |
| Memory recall during the intervention | 13.13 (13.72) | 74.55 (18.53) | 44.63 (26.84) |
| Cumulative Tetris score | 17180 (11500) | 13290 (9210) | n/a |
| Intrusive memory |  |  |  |
| Diary intrusions | 3.69 (2.98) | 4.10 (4.01) | 3.10 (2.34) |
| Diary compliance | 74.67 (11.16) | 79.00 (12.25) | 79.20 (12.30) |
| IES (intrusion subscale) | 7.10 (4.72) | 6.91 (4.37) | 7.49 (4.34) |
| PCL-5 (re-experiencing subscale) | 2.37 (1.77) | 1.66 (1.80) | 2.17 (1.76) |
| IPT | 2.23 (1.79) | 2.48 (2.60) | 2.30 (2.40) |
| Diary intrusions ^b^ |  |  |  |
| Intrusion distress | 2.38 (1.59) | 2.37 (1.99) | 2.82 (1.74) |
| Intrusion vividness | 5.07 (1.57) | 5.25 (1.96) | 5.36 (1.58) |
| Intrusion unpleasantness | 4.22 (1.98) | 3.98 (2.29) | 4.99 (1.87) |
| Intrusion arousal | 2.86 (1.76) | 2.88 (1.81) | 3.52 (1.37) |
| Intrusion suddenness | 6.61 (1.26) | 6.69 (1.76) | 6.32 (1.58) |

*Note*. BDI-II = Beck-depression inventory-II; STAI-T = State trait anxiety inventory – trait; TEQ = Traumatic Experiences Questionnaire; IES = Impact of Event Scale; PCL-5 = PTSD Checklist; IPT = Intrusion Provocation Test Means (SD) are reported.

^a^ Negative mood is a composite score of sadness, happiness (reversed), horror, fear, anxiety, and depression mood ratings

^b^ Thirteen participants were excluded from the analyses of intrusion distress, vividness, unpleasantness, arousal, and suddenness because they reported no intrusions. The remaining participants per condition were: No-Task Control (*n* = 26), Tetris with Memory Recall Task (*n* = 26), and Tetris without Memory Recall Task (*n* = 25).

**Table S2**

*Baseline Measures and Reaction Times for the Three Groups.*

|  | RIR without Memory Recall Task | RIR with Memory Recall Task | RIR Only |
| --- | --- | --- | --- |
| Baseline |  |  |  |
| Sex: men/women (*n*) | 3 / 12 | 5 / 10 | 4 / 11 |
| Age (years) | 20.93 (1.49) | 20.80 (1.66) | 21.87 (2.30) |
| Reaction times |  |  |  |
| Block 1 | 246.99 (18.00) | 283.69 (43.63) | 249.53 (19.32) |
| Block 2 | 241.43 (13.97) | 264.53 (44.11) | 248.50 (25.83) |
| Block 3 | 249.16 (12.35) | 258.98 (30.82) | 253.16 (30.08) |
| Block 4 | 247.19 (16.49) | 260.95 (30.97) | 259.68 (32.08) |
| Task performance |  |  |  |
| Attention (to the film) | 91.73 (4.15) | 85.93 (5.87) | n/a |
| Familiarity (with the film) (*n*) | 2 | 0 | 0 |
| Memory recall during RIR | 54.93 (24.92) | 70.80 (9.53) | n/a |
